# Polygenic Risk Scores for Pediatric Obsessive-Compulsive Symptoms and their Mediating Effect in Clinically Diagnosed Samples of Obsessive-Compulsive Disorder, Attention-Deficit/Hyperactivity Disorder, Anxiety, Depression, Autism and Tourette syndrome

**DOI:** 10.21203/rs.3.rs-7115885/v1

**Published:** 2025-08-06

**Authors:** Lilit Antonyan, S-M Shaheen, Christie Burton, William Gehring, Noam Soreni, Pamela Falzarano Szura, Julia Bellamy, Usha Rajan, David Rosenberg, Gregory Hanna, Paul Arnold

**Affiliations:** Mathison Centre for Mental Health Research and Education, Hotchkiss Brain Institute, Cumming School of Medicine, University of Calgary, Calgary AB, Canada; Mathison Centre for Mental Health Research and Education, Hotchkiss Brain Institute, Cumming School of Medicine, University of Calgary, Calgary AB, Canada; Department of Neurosciences and Mental Health, Hospital for Sick Children, Toronto, ON, Canada; Department of Psychology, University of Michigan, Ann Arbor MI, USA; Department of Psychiatry and Behavioural Neurosciences, McMaster University, Hamilton, ON, Canada; Department of Psychiatry and Behavioral Neurosciences, Wayne State University, Detroit MI, USA; Department of Psychiatry and Behavioral Neurosciences, Wayne State University, Detroit MI, USA; Department of Psychiatry and Behavioral Neurosciences, Wayne State University, Detroit MI, USA; Department of Psychiatry and Behavioral Neurosciences, Wayne State University, Detroit MI, USA; Department of Psychiatry, University of Michigan, Ann Arbor MI, USA; Mathison Centre for Mental Health Research and Education, Hotchkiss Brain Institute, Cumming School of Medicine, University of Calgary, Calgary AB, Canada

## Abstract

Here, we present the first genome-wide association study of obsessive-compulsive symptoms in a sample of clinically diagnosed pediatric participants and healthy controls. Using a psychiatric questionnaire score as a quantitative trait we conducted a large-scale genetic analysis and ran multiple post-association analyses to investigate the mediating role of obsessive-compulsive symptoms in six comorbid neuropsychiatric disorders. Although no SNPs reached genome-wide significance, we identified suggestive associations on chromosomes 4, 5, 6, 7, 9, 17, 19, and 22. Notable genes mapped to these regions were highlighted, though none met the threshold for multiple testing correction. Further, polygenic risk scoring and Mendelian randomization analyses explored the potential mediating role and genetic disposition of obsessive-compulsive symptoms in obsessive-compulsive disorder, anxiety, attention-deficit/hyperactivity disorder, depression, autism spectrum disorders and/or Tourette syndrome. We found that genetic predisposition for OCS accounts for approximately 2% in individuals with one or more of these six disorders, with a particularly strong mediation effect observed for anxiety disorders. This study underscores the value of examining genetic risk across the symptom spectrum of mental illnesses, rather than relying solely on binary diagnostic categories.

## Introduction

Obsessive-compulsive (OC) symptoms (OCS), characterized by intrusive thoughts and repetitive intentional behaviors are common in children and youth [1]. These symptoms represent the core features of obsessive-compulsive disorder (OCD), and evidence from large community-based studies of children and youth indicates that OCD and related disorders are associated with higher severity scores on continuously distributed measures of obsessive-compulsive symptoms (OCS) in the general population [2]. Many studies report that OCD is highly comorbid and occurs with many other mental disorders such as anxiety (ANX), major depressive disorder (MDD), attention deficit/hyperactivity disorder (ADHD), autism spectrum disorders (ASD) and Tourette syndrome (TS) [3–5]. OCS are also seen in many of these disorders (particularly tic, grooming and autism spectrum disorders) whether or not the individual meets full criteria for OCD.

According to the World Health Organization, OCD is among the ten most disabling conditions globally [6]. First-line treatments for OCD include medication and cognitive-behavioral therapy. Although these treatments have been shown to be efficacious in multiple controlled trials, the response rates are 47–57% [7], indicating that approximately half of children with OCD will continue to experience significant symptoms. The long-term persistence rate of OCD is 41% [8], however, it increases up to 60% when subclinical symptoms are included. OCD negatively impacts quality of life in various aspects, such as education, employment, social life, and familial relationships [9]. Despite increasing evidence from multiple studies, there are many remaining questions regarding the pathophysiology of OC traits, OCD and related disorders. The pathophysiology involves multifactorial complex interplay between neurological abnormalities, genetic predispositions, and environmental factors. Substantial evidence indicates that the neural basis of OCD is dysfunction of cortico-striato-thalamo-cortical (CSTC) circuitry [10]. A number of studies showed that OCD is highly heritable, in particular, twin-based studies of OCS dimensional traits report around 30% heritability in adults and around 47–67% in children [11–15].

OCS may be measured by different standardized questionnaires. One of the best accepted pediatric questionnaires is the Child Behavior Checklist Scale – Obsessive-Compulsive Subscale (CBCL-OCS) which is shown to be highly effective to screen for OCS in children [16][17]. Hudziak et al. described 55% heritability in the largest twin study of CBCL-OCS as a quantitative trait, further identifying that OCS is influenced by additive genetic factors without genetic differences across sex [18]. CBCL-OCS is a parent report questionnaire, derived from the widely used CBCL measure on which the subject is rated on various behavioral and emotional problems, with an equivalent self-report form used in children 12 and over [17]. Hudziak et al. reported that the CBCL-OCS has high sensitivity (92%) and moderate specificity (67%) to distinguish clinical OCD cases compared with healthy controls [18].

Genetic etiology of OCD and OCS remains unclear, although recently progress has been made in identifying potential genetic risk variants. Numerous candidate gene analyses and genome-wide association studies (GWAS) have been conducted to identify the genetic pathophysiology of OCD and OC traits. A number of candidate genes from mainly serotonergic, dopaminergic, and glutamatergic systems were published with mixed findings [11][19][20].

In comparison to candidate genetic studies which focus on selected single variants, GWAS involves scanning common single nucleotide polymorphisms (SNPs) across the whole genome to identify risk variants. The biggest challenge of GWAS is to provide a large enough sample size and test millions of SNPs. To overcome the sample size issue, GWAS data can be utilized for generating polygenic risk scores (PRS). PRS is calculated from the additive effects of multiple genetic variants that contribute to overall genetic risk of the trait. It is assessed using discovery GWAS data from common biobanks and consortiums to measure individual’s genetic liability to the trait [21]. Mendelian randomization (MR) is another useful post-GWAS analysis tool to estimate causal relationships between common genetic variants and traits of interest through a mediator [22][23].

To date, there are multiple GWAS studies on OCD. The first two GWAS studies did not detect any genome-wide significant variants [24][25]. The study by Mattheisen et al. identified a SNP near the gene *PTPRD* to have a suggestive strong association of p-value = 4.13×10^−7^ [25]. Next, a meta-analysis of these two studies including 2 688 cases and 7 037 controls were performed, however, no hits were identified [26].

Later, Burton et al. conducted GWAS on quantitative OC traits, using the Toronto Obsessive-Compulsive Scale (TOCS) [27] as an OCS measure on 15 880 community-based samples from Spit for Science study [2] [28]. rs7856850 SNP on *PTPRD* gene reached genome-wide significance level (p-value = 2.48 × 10^−8^) [2], that was also the closest to the significance locus identified in a previous study by Mattheisen et al. [25]. Recent GWAS on OCD by OCD/Tourette Syndrome Working Group of the Psychiatric Genomics Consortium (PGC) was groundbreaking as it increased the previous sample size threefold, leading to substantially increased power. Strom et al. conducted the analysis on 37 015 cases and 948 616 controls and were able to detect multiple genetic variants that have genome-wide significance. They identified 15 independent genome-wide significant loci, that were located on chromosomes 1, 2, 3, 4, 5, 6, 8, 11, 12, 15, and 21. The post-GWAS gene-based analysis also identified around 80 genes to be significantly associated with OCD. They carried out linkage disequilibrium score (LDSC) regression analysis to show if there is any genetic correlation between OCD and other phenotypes. Psychiatric phenotypes like ANX, MDD, TS, post-traumatic stress disorder, anorexia nervosa, neuroticism, suicide attempt and history of childhood maltreatment were positively correlated with OCD [29].

GWAS on OCS have also been performed using self-report measures from population-based samples [30] [31]. The largest GWAS of OCS, conducted on 33,943 individuals, demonstrates a significant polygenic contribution and reveals shared genetic risk factors with clinically diagnosed OCD [31]. No SNP or gene reached the genome-wide significance. After running LDSC genetic correlation analysis they observed a strong correlation between OCS and OCD, as well as other psychiatric disorders such as ANX, MDD, schizophrenia (SCZ), anorexia nervosa and neuroticism. Moreover, they carried out PRS analysis using their target data against publicly available summary statistics of OCD, MDD, ASD, ADHD, SCZ, and educational attainment. In summary, OCD and SCZ had significant association with OCS [31].

Here, we present a unique study on pediatric samples that are clinically diagnosed with one or more psychiatric disorders. To date, no studies of OCS have been conducted that include clinical samples of individuals diagnosed with OCD and other mental health disorders.

Initially, GWAS was carried out to investigate genome-wide associations of OCS severity measured by CBCL-OCS as a continuous trait. We then tested if the effect of OCS genetic risk markers on OCD, ANX, ADHD, MDD, ASD and TS are mediated through OCS using PRS and MR analysis.

## Materials and Methods

### Participants

Child and youth participants between the ages of 6–25 were recruited. Every participant had a general medical exam by their primary care physician within the preceding year and met the inclusion/exclusion criteria (Supplementary Material 1.1). In addition to these general criteria, for inclusion in this study we specified that 1) “cases” should be clinically diagnosed with one or more mental health disorders, such as OCD, ANX, ADHD, ASD, MDD and/or TS; 2) “healthy controls” should have no lifetime diagnosis of any mental disorder, no history of intrusive thoughts, repetitive behaviors, or mental rituals meeting according to DSM-5 criteria [32]. More detailed descriptive statistics on samples are in Table S1 and Table S2.

For genotyping purposes saliva and/or blood were collected to extract DNA samples. The samples were obtained from different sites including: 1) University of Michigan, MI, and 2) Wayne State University, MI, and 3) SickKids Hospital in Toronto, ON. Predominantly 661 European ancestry participants were included, as we had to exclude other ancestries due to the difficulty of controlling heterogenous ancestries. All 661 samples underwent the clinical assessment process. Of this sample, 446 individuals were clinically diagnosed “cases” and 215 were “healthy controls”. Four different genome-wide arrays were used for genotyping purposes. Illumina Multi-Ethnic Global-8 kit (MEG) array, Illumina Omni 2.5–8 kit, PsychChip Illumina microarray, and Human Core Exome kit arrays were used for genotyping (Supplementary Material 1.2). The markers from different arrays were merged and imputed before running through quality control (QC) analysis.

### Genome-Wide Association Analysis

To increase the marker coverage, SNP imputation analysis was carried out on the Michigan Imputation Server (MIS) via Minimac4 software using 1000 Genome project phase 3v5 as the reference [33]. Eagle v2.4 was implemented for phasing. Samples underwent rigorous quality control both prior to and following imputation. The resulting output files were then processed through comprehensive QC filters, as recommended by the MIS team [33][34]. QC analysis before running GWAS was conducted using standard methods with PLINK v2.00 [35]. The major QC steps for individuals and genetic variants are explained elsewhere [36][37]. Genotyping call rates > 3%, minor allele frequency (MAF) of 1%, INFO (information metric) scores of 0.3, Hardy Weinberg equilibrium < 10^−6^, second degree of relatedness were selected as QC thresholds for this study (Supplementary Material 1.3.2).

Population stratification analysis was applied using the multidimensional scaling (MDS) approach (Figure S4) [38]. The first four components (PC1-PC4) were used as covariates for the study. Additional utilized covariates were age, age^2^, and biological sex.

All the data cleaning and standardization was performed using R software packages [39] and/or IBM SPSS Statistics, Version 26. Post-imputation quantitative association analysis using generalized linear model (GLM) was conducted via PLINK v2.00 [35]. Genes that were near the genome-wide significant loci were mapped via LocusZoom [40]. We also checked if identified genes are differentially expressed in brain regions using GTExPortal database [41]. The traits and phenotypes that were previously reported in literature to be associated with identified genes were investigated accessing “Ensembl” public database [42].

### Gene-Based Analysis

Gene-based and gene-set analyses were carried out using Functional Mapping and Annotation (FUMA) technique [43]. FUMA SNP2GENE tool was used for identifying genes that have functional consequence. FUMA also runs via the Multi-marker Analysis of GenoMic Annotation (MAGMA) v1.10 tool [44]. MAGMA uses GWAS summary statistics and is based on a multiple regression model to account for LD between the variants. Gene positional mapping was done via 1000 Genomes phase 3 reference panel. To correct for multiple testing a Bonferroni P-value < 0.05/n threshold was set, where n was the number of annotated genes.

Gene-set analysis was conducted based on the genes identified from the previous step. This was done using MAGMA v1.10 [44] and FUMA GENE2FUNC tool [43]. It involves the set of curated gene sets and GO terms obtained from Molecular Signatures Database (MsigDB.v7.5.1) [45]. To be considered significant gene-sets should pass the Bonferroni correction P-value < 0.05/n threshold, where n is the number of gene-sets.

### OCS as mediator

#### Heritability estimate

Heritability estimate (h^2^) was assessed through LD regression score (LDSC) where genetic heritability is assumed. LDSC was assessed using bigsnpr R package by Privé et al. [46]. Due to the small sample size, LDSC was calculated using reference LD matrix obtained from HamMap3 + which is an extended set of HapMap3. HapMap3 + reference data is well imputed and covers the whole genome.

#### Polygenic Risk Score Analysis

PRS shows the collective significance of multiple SNPs using GWAS data. Moreover, mental disorders are inherently complex, with genome-wide associations reflecting a highly polygenic architecture; thus, exploring polygenicity and PRS may offer greater predictive utility [21] [47] [48]. To generate polygenic risk scores (PRS), we used our clinically based samples as the target dataset, while the base dataset consisted of summary statistics from the large-scale cohort provided by the PGC consortium [49] [50]. In other words, we executed PRS analysis using our OCS samples as target data, and six mental disorders as six different base data. This enabled us to test how the genetic disposition to OCS may predict the risk of having one or more of the six mental disorders.

GWAS summary statistics of OCD [29], ANX [51], ADHD [52], MDD [53], ASD [54], and TS [55] were accessed from different working groups of the PGC consortium (Supplementary Material 1.4.1).

There are multiple available techniques currently recommended for carrying out PRS analysis such as LDpred2 [56], PRSice2 [57], PRS-CSx [58], Lassosum [59], etc. We used the LDpred2 method that is based on the Bayesian shrinkage model [56][60]. LDpred2 has been widely used and has challenges and limitations which we addressed thoroughly [56]. LDpred2 allows the inclusion of an LD matrix that was obtained from the HapMap3 + reference genome. It also accounts for heritability explained by the SNPs (LDSC). LDSC should be bigger than 0.05 to proceed with meaningful PRS analysis. LDpred2-auto was selected as it automatically estimates sparsity P and LDSC scores, and further validation set is not required. Analysis was conducted using *bigsnpr* R package [46] adopting provided script by Privé et al. [56].

To summarize, PRS analysis using our target samples were conducted using six different base datasets: OCS, OCD, ADHD, Anxiety, Autism, MDD, and TS (Supplementary Material 1.4.1).

In addition to the cross disorder PRS analysis, we also performed PRS using C + T PRSice2 method [57] using an OCS study by Strom et al. as base data to find the association of PRS to OCS severity. The GWAS summary statistics were obtained from the largest OCS GWAS study that included around 34 000 participants [31]. At this step we were able to identify the best PRS model that can identify predictability of OCS. This also enabled us to identify the SNPs that contribute to the high polygenicity risk of OCS.

#### Mendelian Randomization

**Figure 1:**
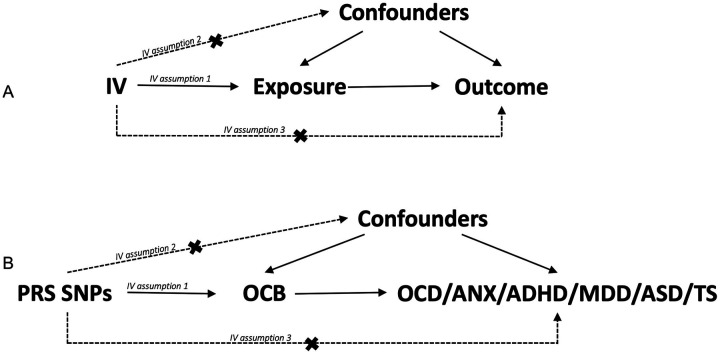
A general and specific case of MR. A) a general case of MR, where it estimates whether the correlation between exposure and outcome is causal in the presence of confounders. B) a specific case of MR adapted to our analysis, where IVs are SNPs contributing to PRS, OCS is the exposure, and 6 disorders are the outcomes. Confounders are the covariates that were also used in the GWAS and PRS analysis, such as biological sex, ethnicity (PC1-PC4), and age. The *IV assumption 1* is obtained from our target GWAS summary statistics. IV assumption 3 is very crucial and to test that PGC summary statistics from 6 disorders were used.

The SNPs that contribute to high risk from the PRS C + T method were utilized as instrumental variables (IV) to run a MR analysis to determine if there is any causal interference between identified polygenic risk loci and the disorders where OCS is the exposure (or assumed mediator) (Fig. 1). The choice of IVs from C + T method also assumes that the SNPs are uncorrelated.

MR is an ultimate epidemiological technique that assesses relationships between risk factors and traits to show if the correlation between them may be causal [22][23]. Three main assumptions for IVs are explained elsewhere [22][23]. The MR estimates are considered to show a pleiotropic effect under the instrument strength independent of direct effect (InSIDE) assumption [22][23]. Cochran’s Q values were calculated to detect the heterogeneity bias and invalid IVs.

Two-sample MR analysis using an inverse-variance weighted (IVW) approach was applied. IVW is the most widely used approach that combines the estimates from multiple genetic instruments into a single estimate of causal effect of an exposure [61][62].

MR IVW analysis was carried out using *MendelianRandomization* R package [63]. Next, the most influential observations were detected using Cook’s distance and removed via leave-one-out approach [64] [65]. Additionally, MR-PRESSO was applied to test the validity of MR test and check for horizontal pleiotropy bias [66][67]. Outliers were removed and we reran IVW MR on the remaining IVs for more reliable results. More detailed methodology can be found in Supplementary Material 1.5.

## Results

### Samples

GWAS quality control analysis was first performed to ensure high quality genotype-phenotype data (Figure S2, S3). A total of 95.7% of variants passed the missingness thresholds. MAF later excluded ~ 45% of markers that had frequency smaller than 0.01. Next, 99.9% of remaining markers passed HWE threshold. A total of 38 individuals were excluded from the analysis due to high missingness, high heterozygosity rate, relatedness and population stratification filters. Further, another 36 samples have either missing phenotype, or missing covariate information that were excluded as well.

Overall, after data cleaning, standardizing, and QC, high quality genotype-phenotype data included 587 samples and 1 751 493 markers.

During the SNP imputation step around 45 million variants were *imputed* (96% imputation rate) (Figure S2). Only 20% of the imputed SNPs passed the post-imputation QC filters of INFO > 0.3 and MAF > 0.01 (Figure S3). In total 6 047 748 SNPs remained for further association analysis.

### Genome-Wide Association Analysis

**Figure 2:**
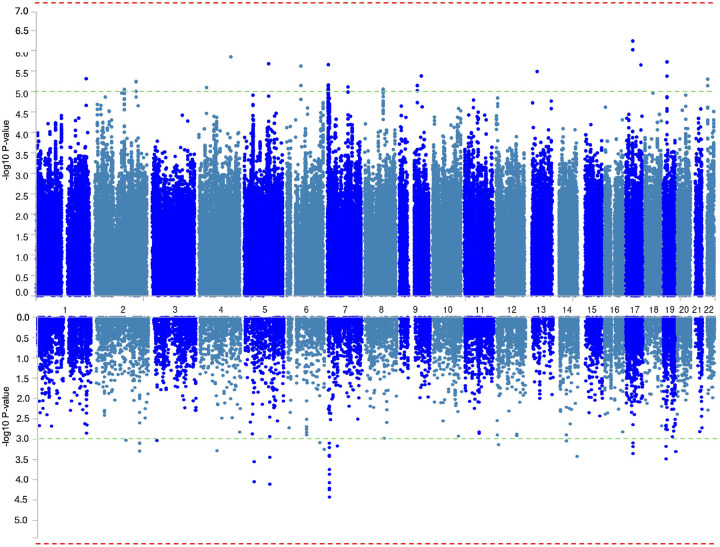
Miami plot of the GWAS summary statistics. Miami plot of the GWAS summary statistics of 587 samples using OCS (CBCL-OCS) as quantitative trait. Upper panel is the SNP-based association results and lower panel is gene-based association analysis. Dashed red lines are strict p-value thresholds of 5×10^−8^ (upper panel); and 2.8×10^−6^ (lower panel). Dashed green lines are suggestive significance p-value thresholds of 1×10^−5^ (upper panel); and 1×10^−3^ (lower panel).

A GWAS of OCS using CBCL-OCS as a quantitative trait was run on 587 samples and included around 6 million markers to test associations. A Miami plot of the SNP-based (upper panel) and gene-based (lower panel) association results are shown in Fig. 2. No genomic inflation was detected, QQ plots of the SNP-based (λ = 0.993) and gene-based (λ = 1.03) association results are in Figure S5. As expected, due to the small sample size no significant variants passed the strict Bonferroni p-value thresholds: SNP-based association p-value < 5×10^−8^ (Fig. 2 upper panel); gene-based association p-value < 0.05/18 055 = 2.8×10^−6^ (Fig. 3.2 lower panel). Consequently, we used suggestive significance p-value thresholds of 1×10^−5^ for SNP-based (Fig. 2 upper panel) and 1×10^−3^ for gene-based tests (Fig. 2 lower panel) to identify top associations. Table 1 shows top genome-wide significant SNPs along with their alleles, effect sizes, MAF, INFO scores, and genes that are at the nearest distance to them. SNPs from chromosomes 4, 5, 6, 7, 9, 13, 17, 19, and 22 have suggestive significant association with quantitative OCS as measured using the CBCL-OCS (Table 1). rs150013080 is directly mapped to *AP2B1* gene, rs111805629 to *NR3C2*, *rs424608* to *IL12RB1*, rs419939 to *TICAM2*, rs139834775 to *GRID2IP*, rs9567245 to *ENOX1*, rs77498727 to *PLPPR1*, the remaining three SNPs were in the regions of pseudogenes or lincRNA (Table 1) and current information on these SNPs and genes are very limited. A closer look on the identified loci is visualized in Figure S6.

No genes or gene sets passed the Bonferroni-corrected P-value thresholds. Top 10 suggestive significant genes were from these 3 loci: 7p22.1 locus with *GRID2IP, CYTH3, C7orf26, DAGLB, ZNF12* and *KDELR2* genes, 5q22.1 locus with *TICAM2* gene, 5p13.1 with FBXO4 and C5orf51 genes (Fig. 2). The results of the gene-set analysis are reported in Table S4.

Most of the identified genes from SNP-based and gene-based GWAS were strongly expressed in brain according to GTExPortal database [40]. Specifically, *AP2B1, GRID2IP, PLPPR1*, and *DAGLB* were more differentially expressed in brain than in other tissues. Followed by *NR3C2, ENOX1, SLC7A4*, and *ZNF12* genes that exhibited differential expression in brain as well as in other organs.

### Polygenic Risk Score

**Figure 3:**
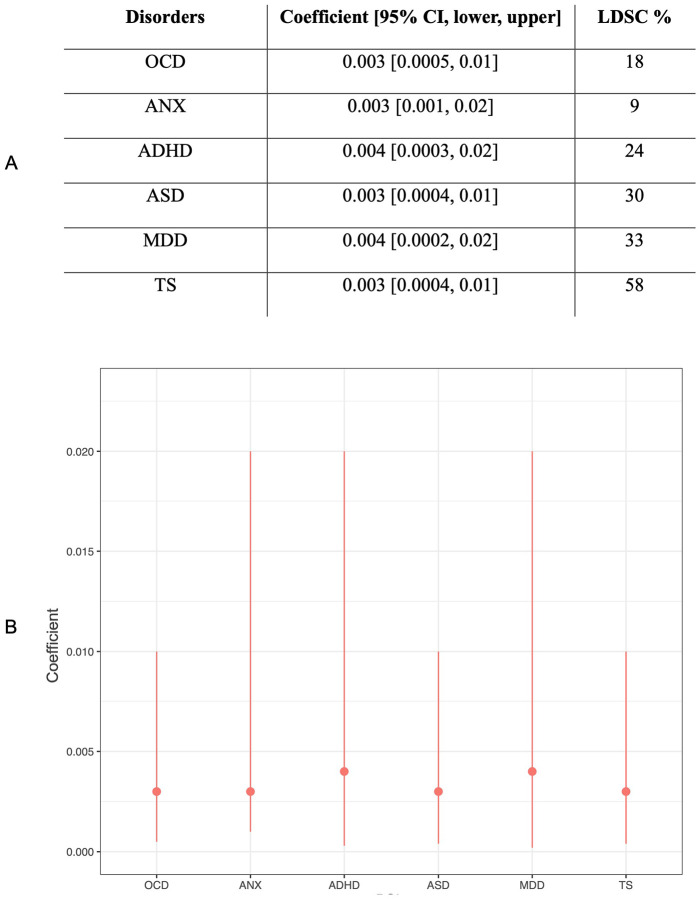
Results after LD-pred2-auto PRS analysis. The coefficient represents the partial correlation between the phenotype and PRS along with their 95% CI lower (2.5%) and upper (97.5%) bounds. LDSC score is computed using HapMap3+ reference matrix. Proportion of the variance explained (Coefficient) is under 0.02. A) shows the result of 6 disorders partial correlation with lower (0.25%) and upper 0.975% CI (95%) bounds, and their LDSC scores. B is the visual representation of the partial correlation values with their lower (0.25%) and upper 0.975% CI (95%) bounds (y axis) for mentioned 6 disorders.

The estimated SNP heritability of OCS from LDSC was ~ 6%. The relatedness matrix included ~ 63% of the genotyped markers. PRS analysis was run using our target OCS data (587 samples) against six disorders: OCD (23 493 cases/1 114 613 controls) [29], ANX (18 186 cases/17 310 controls) [51], ADHD (38 691 cases/186 843 controls) [52], MDD (170 756 cases/329 443 controls) [53], ASD (18 382 cases/27 969 controls) [54], and TS (4 819 cases/9 488 controls) [55]. For all six tests around 1 million SNPs were matched between our target sample, the HapMap3 + dataset, and corresponding summary statistics (Figure S7). Figure 3 shows the results of the cross-disorder PRS analysis that were carried out using LDpred2-auto. The partial correlation coefficients are consistent, some of them showing higher median, and others higher upper bound indicating that there is a significant although weak correlation (< 2%) between PRS of OCS and all six disorders.

### Mendelian Randomization

A total of 356 SNPs contributing to the PRS score were obtained from the C + T tool PRSice2 [57]. The best model for PRS fit between our OCS target data (587 samples) and OCS base data (33 943 samples) [31] had the p-value of 0.05 (Figure S10). To test if OCS may act as a mediator between genetic markers and disorder outcome (Fig. 1B), IVW MR was carried out on the selected and pruned 500 IVs. Table 2 shows the results of IVW MR after Cook’s distance and MR-PRESSO outlier filtering for all six disorders.

**Figure 4:**
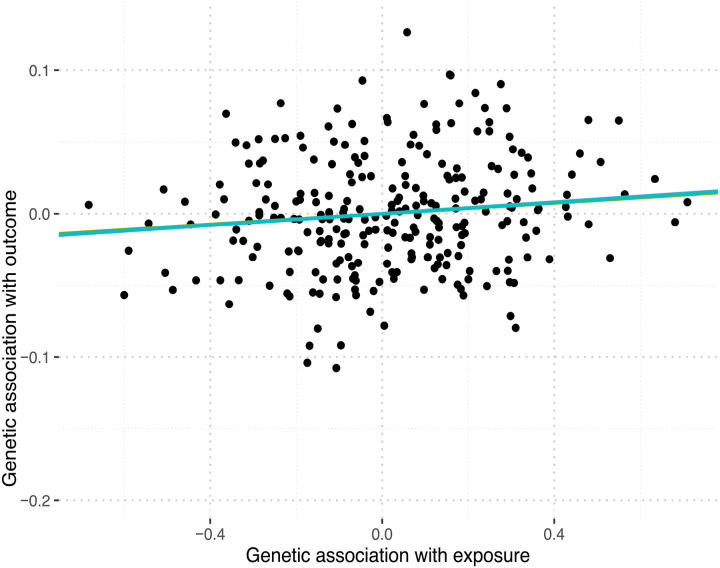
IVW MR estimate for ANX. The causal relationship between the genetic association with ANX and genetic association with OCS is positive meaning that the increase of OCS may be responsible for ANX diagnosis.

The causal estimate for ANX was significant with p-value = 0.04. Cochran Q statistics did not detect any heterogeneity bias between the IVs of ANX (Cochran’s Q = 275 on 279 degrees of freedom), ASD (Cochran’s Q = 439 on 464 degrees of freedom), and TS (Cochran’s Q = 425 on 459 degrees of freedom) (Table S5). Additionally, global test results from MR-PRESSO indicate that horizontal pleiotropy bias was detected for only OCD (p < 0.001) and MDD (p = 0.006) (Table S5). Figure 4 shows the tendency of causality interference between OCS and ANX which exhibits positive causal relationship. Thus, it is assumed that SNPs-outcome effects are influenced through OCS exposure.

## Discussion

This study represents the first genome-wide study employing CBCL-OCS as an OCS quantitative trait in clinically diagnosed “cases” (diagnosed with OCD, ANX, ADHD, MDD, ASD, and TS), and “healthy controls” free of mental illness. All participants received detailed feedback including semi-structured interviews to confirm diagnoses. However, a limitation specific to the ASD participants is that they did not receive the Autism Diagnostic Interview (ADI) or the Autism Diagnostic Observation Schedule (ADOS) which are considered gold standard for individuals with these diagnoses.

No SNPs reached the Bonferroni corrected significance threshold of 5×10^−8^, likely due to limited statistical power given the small sample size for both SNP-based and gene-based GWAS.

After the SNP-based GWAS we identified top SNPs with the lowest p-values and their nearest gene (Table 1). Two of the top suggestive SNPs: rs150013080 and rs145688353, are located in the region of *AP2B1* (adaptor-related protein complex 2, beta 1) gene (Figure S6 A). *AP2B1* is a protein coding gene implicated in cerebellar degeneration [68] [69] and plays a crucial role in synaptic neurotransmission [70]. Additionally, *AP2B1* is associated with cognitive ability and educational attainment [71].

Another top SNP is rs111805629 with the nearest *NR3C2* (nuclear receptor subfamily 3, group C, member 2) protein coding gene (Figure S6 E). In GWAS studies, several SNPs of *NR3C2* are associated with ASD [72–74] hippocampal volume [75] and Alzheimer’s disease [76]. Other suggestive hits in our data included SNPs from chromosome 5q22.3 including rs419939 SNP located in *TICAM2* (toll-like receptor adaptor molecule 2) gene (Figure S6 D) which is involved in signaling pathways [77][78] and inflammatory responses [79]. Another highly prevalent locus is 7p22.2 which has several strong associations including rs139834775 (Figure S6 C). This locus maps to *GRID2IP* (glutamate receptor, ionotropic, delta 2 (Grid2) interacting protein) gene, which interacts with *GRID2* gene expressed in Purkinje cells and plays a major role in synaptogenesis and synaptic plasticity [80]. Glutamate neurotransmission is highly prevalent in many mental disorders such as OCD [81], ANX [82], ADHD [83], MDD [84], ASD [85], TS [86], and others. Notably, the loci on chromosomes 5q22.3 and 7p22.2 were also strong hits in gene-based GWAS, further suggesting the involvement of signalling pathways in mental disorders. Another interesting gene that was mapped to a strong signal, rs77498727, is *PLPPR1* (lipid phosphate phosphatase-related protein type 1) (Figure S6 G) which is strongly expressed in brain and plays a crucial role in neuronal plasticity [87][88]. Additionally, some SNPs of *ENOX1* (ecto-NOX disulfide-thiol exchanger 1) gene where the identified rs9567245 SNP is located (Figure S6 F) have been linked to MDD [89] and schizophrenia [90]. Furthermore, the PGC cross-disorder study on eight disorders (OCD, ADHD, ASD, MDD, TS, anorexia nervosa, bipolar disorder, and SCZ) identified SNPs of *ENOX1* gene among the top SNPs [4].

The genes with lowest p-value from gene-based GWAS have strong hits in three regions on chromosome 7 and 5: *GRID2IP, CYTH3, C7orf26, DAGLB, ZNF12* and *KDELR2* genes from chromosome 7; *TICAM2, FBXO4* and *C5orf51* genes from chromosome 5. As mentioned above, SNPs from GRID2IP and TICAM2 overlap with the SNP-based GWAS hits. Additional notable genes were *DAGLB, KDERL2, ZNF12* and *C7orf26*. A GWAS study on ADHD indicated that *DAGLB* and *KDERL2* have significant associations with inattention symptoms of ADHD in family-based samples [91]. Moreover, a genome-wide interaction study found that SNPs mapped to *KDELR2, CYTH3* and *C7orf26* genes were associated with body mass index (BMI) [92]. Another GWAS identified *ZNF12* as being associated with BMI [93]. We also observed that most of these genes, specifically *AP2B1, GRID2IP, PLPPR1*, and *DAGLB, NR3C2, ENOX1, SLC7A4*, and *ZNF12*, were differentially expressed in brain tissues.

The results of PRS analyses indicate that genetic liability of OCS is correlated with the risk of developing mental disorders where OCS are prevalent. Although the partial correlation of OCS with PRS was significant, it was relatively weak (< 2%) (Fig. 3), suggesting that the genetic predisposition of OCS might be limited. The current DSM-5 diagnosis is based on broad range of symptomology therefore it results in heterogenous genetic profiles which is a predominant pattern in other psychiatric studies as well [94] [95].

The central assumption of PRS is that the variants are independent and have a normal (Gaussian) distribution. Thus, multiple testing and overfitting can pose significant limitations to any PRS analysis [21] and contribute to weak findings in our analysis. To mitigate these issues, LDpred2-auto approach was selected as it automatically estimates sparsity P and has shown better prediction ability compared to other methods. It may underperform in certain situations, especially when multi-ancestral samples are included [56].

The IVs for MR were chosen from a standard PRS C + T method such as PRSice2, which was used to determine the proportion of SNPs responsible for the polygenicity of OCS. To address the issue of correlated variants, LD clumping and pruning of the samples were carried out.

MR analysis was conducted to better understand the genetic contribution of OCS and its possible correlations with other mental disorders. It is important to note that MR analysis relies on several assumptions that must be met in order to report meaningful results. To ensure this, we tested for potential biases such as horizontal pleiotropy using the MR-PRESSO Global Test and IV heterogeneity using Cochran’s Q estimate. No heterogeneity bias was found among the IVs for ANX, ASD and TS tests (Table S5). OCD and MDD exhibited horizontal pleiotropy bias (Table S5), indicating that while OCS might be a potential exposure for OCD and MDD, other intermediate factors may also contribute to OCD diagnosis.

Among the six disorders studied, causal pleiotropic effects were observed between OCS SNPs and ANX, with no bias detected in the ANX test. Thus, we infer that the polygenic risk variants of OCS have a positive causal effect on ANX, and this effect is mediated by OCS. Ultimately, no causal relationship was detected with OCD or any other disorder. This can be attributed to the nature of the OCS phenotype, which was assessed via CBCL-OCS questionnaire. Indeed, the eight items included in CBCL-OCS [17] investigate various OC and anxious behavioral problems and might equally be associated with both OCD and ANX [17][96]. Additionally, over 30% of our samples were diagnosed with ANX. While preliminary, the results of the MR tests shed the light on the mediating effects of OCS. A choice of an alternative scale such as TOCS [27] might have addressed this challenge; however, these were not available for the majority of our participants.

The results of the current study should be considered with several limitations in mind. Unfortunately, one of the most common limitations of the study is that our sample was almost entirely of European ancestry, which limits the generalization of findings to other ethnic groups. Many ongoing studies aim to address this by including samples from multiple ethnic backgrounds.

Another substantial limitation of association studies is that they typically only include common variants. Further research using next-generation sequencing methods such as whole exome sequencing [97] or whole genome sequencing should be conducted. To identify the contribution of rare variants, our group is currently conducting a whole genome sequencing study of selected samples that overlap with the dataset.

Although the minimal requirement to run a GWAS is over 100 samples, it is much more effective with hundreds of thousands of samples [98]. We addressed the challenge of sample size by conducting postGWAS tests, such as PRS and MR analyses, using much larger samples from PGC consortium [54]. Moreover, we collaborate with PGC Working Groups to contribute to a larger sample size to mitigate this challenge.

MR also has several inherent limitations, such as the MR assumptions and selection of uncorrelated IVs [65][66][69]. We addressed these limitations by checking for potential biases of six MR tests.

We demonstrated a weak but significant causal relationship between OCS and ANX. Though the results are in need of replication, they could set the stage for conducting similar analyses in larger samples to investigate mediating effects of OCS for ANX and other comorbid disorders. This study emphasizes the importance of observing the genetic disposition of symptom spectrum of the mental illness rather than relying solely on binary diagnoses.

## Supplementary Material

Supplementary Files

This is a list of supplementary files associated with this preprint. Click to download.

- FigureS1.jpg
- FigureS2.jpg
- FigureS3.jpg
- FigureS4.jpg
- FigureS5.jpg
- FigureS6.jpg
- FigureS7.jpg
- FigureS8.jpg
- FigureS9.jpg
- FigureS10.jpg
- SupplementaryMaterial.docx

## Figures and Tables

**Table 1:** Top 10 of GWAS variants. Top 10 of suggestive genome-wide significant variants identified after association analysis of OCS. Nearest genes to the top variants are identified using gene mapping techniques.

Variant	rsID	Alleles	P-Value	Beta	MAF	INFO	Nearest Gene
17:33961713	rs150013080	C/T	6×10^− 7^	3.9	0.02	0.82	*AP2B1*
4:149062985	rs111805629	C/T	1.4×10^− 6^	2.7	0.04	0.79	*NR3C2*
19:18142423	rs424608	T/C	1.9×10^− 6^	1.3	0.2	0.92	*IL12RB1*
5:114926421	rs419939	T/C	2×10^− 6^	1.1	0.3	0.79	*TICAM2*
7:6575582	rs139834775	G/A	2.2×10^− 6^	2.8	0.03	0.88	*GRID2IP*
17:71773592	rs73343491	T/C	2.3×10^− 6^	1.7	0.1	0.86	*LINC*
6:66903215	rs143062223	A/G	2.4×10^− 6^	4.8	0.011	0.72	*NUFIP1P1*
13:44251688	rs9567245	C/T	3.3×10^− 6^	2.3	0.05	0.7	*ENOX1*
9:104072782	rs77498727	C/T	4.2×10^− 6^	4.4	0.011	0.7	*PLPPR1*
22:21395784	rs6519739	T/C	5×10^− 6^	1	0.4	0.72	*SLC7A4*

**Table 2:** Results of MR IVW analysis. Three p-values are reported where P_IVW_ represent the p-value after running first round of IVW; P_Cook’s dist_ is the p-value of IVW after removing the influential outliers based on Cook’s distance; P_MR−PRESSO_ represents the last round of IVW P-value where outliers are removed after MR-PRESSO tests.

Outcomes	MR Estimate (SE)	P_IVW_	P_Cook’s dist_	P_PRESSO_	N of IVs
OCD	0.004 (0.002)	0.14	0.09	0.09	466
ANX	0.02 (0.01)	0.1	0.038*	0.037*	280
ADHD	−0.002 (0.002)	0.45	0.93	0.93	447
ASD	0.003 (0.003)	0.25	0.43	0.41	465
MDD	0.002 (0.002)	0.33	0.3	0.3	416
TS	−0.002 (0.005)	0.3	0.26	0.23	460
